# Transformative Advances in Vascular Surgery: Five Decades of Innovation in the Management of Aortic, Carotid, and Peripheral Arterial Disease

**DOI:** 10.1002/wjs.70421

**Published:** 2026-05-16

**Authors:** Ghaleb A. Darwazeh, William Q. Duong, Ramola M. Panchal, Ahmed M. Abou‐Zamzam

**Affiliations:** ^1^ Division of Vascular Surgery Loma Linda University Health Loma Linda California USA

**Keywords:** aortic therapy, carotid artery disease, peripheral arterial disease, surgical innovation

Over the past five decades, vascular surgery has undergone extraordinary transformation, driven by advances in diagnostic modalities, medical therapy, and procedural innovation. The field has evolved from one defined primarily by open surgical reconstruction to a highly sophisticated, multidisciplinary specialty encompassing a wide spectrum of vascular pathologies, including venous disease, peripheral arterial disease, aortic disease, carotid disease, and dialysis access. Parallel progress in vascular imaging—including high‐resolution cross‐sectional imaging, intraoperative imaging, and virtual guidance platforms—has enabled increasingly precise diagnosis and facilitated the safe execution of complex interventions.

While all aspects of vascular surgery have been affected, the most profound advances have occurred in the management of aortic, carotid, and peripheral arterial disease. These domains have witnessed rapid innovation in endovascular technology, refinement of surgical techniques, and the emergence of evidence‐based medical therapy, collectively transforming treatment strategies and patient outcomes.

The management of complex aortic pathology, in particular, has been revolutionized by the development of endovascular technologies. Early experiences with endovascular aneurysm repair (EVAR) demonstrated the feasibility of minimally invasive approaches and paved the way for increasingly complex interventions, fundamentally altering the risk profile of aortic surgery [1]. Compared with traditional open repair, EVAR reduced perioperative morbidity and mortality, particularly in older and higher risk patients, and established a platform upon which subsequent innovations in device design and procedural strategy have been built [2]. These early limitations in device design and anatomic applicability, particularly in patients with short aneurysm necks or renal and or visceral branch vessel involvement, served as a major impetus for the development of more advanced endovascular technologies.

The development of fenestrated and branched endografts represented the next major step forward in extending endovascular therapy to patients with complex aortic anatomy. These technologies allow for the incorporation of visceral and renal vessels into the repair, enabling treatment of juxtarenal, pararenal, and thoracoabdominal aneurysms that were previously only amenable to open surgery [3, 4, 5, 6]. In parallel, physician‐modified endografts (PMEGs) emerged as a flexible and pragmatic solution in urgent settings when off‐the‐shelf devices were not immediately available, as well as in patients whose anatomy was not compatible with either custom‐made or commercially available devices [7, 8]. In many centers, PMEGs provided a critical bridge in the absence of commercially available branched or fenestrated devices, allowing experienced operators to extend endovascular therapy to anatomically complex cases that would otherwise require open repair. Contemporary series have demonstrated that these approaches can achieve favorable technical success and acceptable perioperative outcomes, particularly in high‐risk populations [9, 10].

In addition to physician‐modified endografts, in situ laser fenestration has emerged as a valuable adjunct in the endovascular management of complex aortic pathology, particularly in urgent or emergent settings [11]. This technique allows for the creation of fenestrations within standard endografts to preserve branch vessel perfusion, enabling rapid treatment when custom‐made or branched devices are not readily available. Its application has been described in the management of thoracoabdominal aneurysms and aortic arch pathology, where timely revascularization of critical vessels is essential. Contemporary studies, including systematic reviews, multicenter analyses, and large single‐center experiences, have demonstrated that in situ laser fenestration is a feasible and effective technique with high technical success rates and acceptable early and midterm outcomes, even in complex and emergent cases [12, 13, 14]. Long‐term series, including extensive institutional experiences, have further demonstrated durable results with sustained branch vessel patency and low rates of fenestration‐related complications [15]. Although technically demanding and highly operator‐dependent, in situ fenestration provides a versatile option that further expands the endovascular armamentarium in high‐risk or time‐sensitive clinical scenarios.

More recently, the advent and regulatory approval of multibranched devices such as the thoracoabdominal branch endoprosthesis (TAMBE) have marked a pivotal milestone in the evolution of aortic therapy. These devices offer a standardized, off‐the‐shelf solution for total endovascular repair of thoracoabdominal aortic aneurysms, reducing the need for extensive customization while maintaining the ability to preserve critical branch vessel perfusion [8, 16]. Early and midterm outcomes from multicenter experiences suggest that these devices can achieve high technical success with acceptable morbidity, further expanding the population eligible for endovascular repair [16, 17].

Parallel advances have occurred in the endovascular management of aortic arch pathology, historically one of the most challenging domains in aortic surgery. Initial hybrid approaches, combining supra‐aortic debranching with endovascular exclusion, provided an important bridge toward less invasive therapy [18]. More recently, the development of branched thoracic endografts designed for proximal deployment in the ascending aorta has enabled fully endovascular treatment of complex arch aneurysms and dissections [19, 20]. These approaches have the potential to reduce the physiologic burden of surgery while maintaining cerebral perfusion, addressing one of the principal limitations of earlier open and hybrid techniques. Although long‐term durability data are still evolving, early results are promising and suggest a growing role for total endovascular arch repair in appropriately selected patients [21].

These cumulative advances have been facilitated not only by device innovation but also by significant improvements in imaging, procedural planning, and intraoperative guidance. High‐resolution imaging, three‐dimensional reconstruction, and image‐fusion technologies have enabled increasingly precise execution of complex endovascular procedures, further enhancing safety and expanding the scope of treatable disease. As device technology advances, the scope of treatable pathology via endovascular approaches continues to expand.

A similarly transformative evolution has occurred in the management of carotid atherosclerotic disease over the last half century. Indeed, carotid disease has been one of the most well‐studied vascular pathologies given its potential to offset the high morbidity and mortality of cerebrovascular disease. Starting with the first randomized controlled landmark trials in the 1990s, carotid pathology began to be better understood in terms of its different presentations and outcomes based on plaque morphology, degree of stenosis, and symptom presentation [22, 23, 24]. At the same time, there were major technological advancements in endovascular interventions with the advent of stents, distal embolic protection devices, and flow reversal for neuroprotection. Medical therapy was also being refined during this period, alongside increasing public awareness of risk reduction through smoking cessation and adoption of healthier lifestyles. All of these concurrent advances in prevention, medical management, and surgical techniques have resulted in a contemporary approach that is increasingly individualized to each patient.

To better understand this evolution, it is important to consider the key clinical trials that shaped current treatment algorithms. NASCET and ECST in the 1990s demonstrated that carotid endarterectomy (CEA) was superior to then‐standard medical care alone for patients with symptomatic disease [22, 24]. ACAS in 1995 established that CEA combined with medical management was also superior to medical therapy alone in patients with asymptomatic high‐grade stenosis, with a reported relative risk reduction in stroke of approximately 50% [23]. ACST (2004) further corroborated these findings [25]. Together, these trials established surgical intervention as the standard of care for appropriately selected patients with both symptomatic and asymptomatic carotid stenosis.

With the advent of embolic protection devices, SAPPHIRE (2004) and CREST (2010) demonstrated that carotid artery stenting (CAS) was non‐inferior and a safe alternative to carotid endarterectomy in high‐risk surgical patients [26, 27]. CREST also noted the high risk of periprocedural myocardial infarction in CEA while CAS had higher periprocedural stroke rate, particularly in older patients [27]. Alongside distal embolic protection devices, there were further technological advancements, including dynamic flow reversal during transcarotid artery revascularization (TCAR), which mitigates the risks associated with traversing a heavily diseased aortic arch during transfemoral CAS. The ROADSTER trial (2015) demonstrated that TCAR is a safe and effective treatment for carotid stenosis [28]. Subsequent studies, including ROADSTER 2 and 3, have supported the safety and efficacy of TCAR across broader patient populations [29, 30, 31]. Collectively, these trials demonstrated that carotid stenting represents an effective alternative to surgical intervention for stroke risk reduction in selected patients.

The most recent CREST‐2 trial demonstrated that, for patients with asymptomatic high‐grade carotid stenosis, intensive medical management alone is highly effective, although carotid revascularization may provide additional benefit in selected cases [32]. These results represent a paradigm shift in the management of carotid disease, emphasizing the evolving role of intervention in the setting of increasingly effective medical therapy.

These landmark trials highlight the significant advances in both medical and surgical treatment of carotid stenosis, allowing for optimal, individualized patient management. Future directions will likely focus on improved risk stratification, including the role of plaque morphology, and advanced imaging to better identify patients who may benefit most from intervention.

This shift toward less invasive, evidence‐driven, and patient‐specific care is equally evident in the management of peripheral arterial disease (PAD), which has evolved from a late‐presenting condition managed primarily with open bypass or amputation to one characterized by early diagnosis, advanced endovascular techniques, and comprehensive multidisciplinary limb‐salvage strategies. Despite these advances, chronic limb‐threatening ischemia (CLTI) remains a major global health burden, affecting millions of patients, with substantial risks of amputation and mortality [33].

Modern infrainguinal revascularization can be traced back to the pioneering work of Jean Kunlin, who on June 3, 1948, performed the first successful femoropopliteal bypass using reversed saphenous vein, establishing the foundation of surgical revascularization [34]. Over time, however, minimally invasive endovascular approaches have expanded treatment options and altered the revascularization landscape.

The BASIL trial in 2005 was among the first randomized studies to compare open bypass against angioplasty in patients with CLTI, demonstrating no significant difference in amputation‐free survival [35]. Subsequent technological advances—including improved guidewire systems, stent platforms, plaque modification devices, and drug‐coated balloon technology—have further enhanced the feasibility and durability of endovascular interventions [36]. More recent trials, including LIFE‐BTK and DISRUPT PAD III, have demonstrated the safety and efficacy of emerging technologies such as resorbable scaffolds and intravascular lithotripsy [37, 38].

These advances prompted a re‐evaluation of revascularization strategies, culminating in contemporary randomized trials such as BEST‐CLI and BASIL‐2 [39, 40]. In BEST‐CLI, patients with suitable single‐segment saphenous vein experienced superior limb‐related outcomes with surgical bypass compared with endovascular therapy [39]. In contrast, BASIL‐2, which focused on infrapopliteal disease, demonstrated improved amputation‐free survival with an endovascular‐first approach, driven largely by differences in mortality [40]. Together, these findings underscore that optimal revascularization is highly dependent on anatomy, conduit availability, and patient‐specific risk, rather than a uniform treatment paradigm.

Advances in medical therapy have been equally transformative. The VOYAGER‐PAD trial demonstrated that low‐dose rivaroxaban in combination with aspirin reduces major limb and cardiovascular events following revascularization [41]. Similarly, statin therapy has been shown to significantly reduce major vascular events in patients with PAD [42].

There have also been important advances in diagnosis and surveillance, with increasing emphasis on physiologic measures of limb perfusion, enabling more accurate risk stratification and longitudinal management. Toe pressures and toe‐brachial indices enable assessment in patients with heavily calcified vessels, while transcutaneous oxygen measurements have been validated for stratifying wound healing potential [36, 43]. More recently, pedal acceleration time has emerged as an additional noninvasive tool for evaluating limb perfusion [44].

Perhaps the most important evolution has been conceptual. PAD is no longer managed as an isolated, symptom‐driven surgical condition, but rather as a chronic, systemic disease requiring coordinated, multidisciplinary care. Vascular medicine, endocrinology, wound care, infectious disease, and surgical subspecialties all play integral roles in optimizing outcomes [45, 46].

The convergence of technological innovation, high‐quality clinical evidence, and evolving medical therapy has shifted vascular surgery toward less invasive, more effective, and increasingly individualized care. These advances have not only improved procedural outcomes but have fundamentally redefined the scope of the specialty.

Looking forward, continued progress will depend on further integration of advanced imaging, device innovation, and data‐driven decision‐making, as well as a sustained emphasis on prevention, early diagnosis, and longitudinal care. As the field continues to evolve, vascular surgery is uniquely positioned at the intersection of technology and medicine to deliver increasingly precise, patient‐centered care across the full spectrum of vascular disease.

## Author Contributions

**Ghaleb A. Darwazeh:** conceptualization, writing – original draft, writing – review and editing, supervision. **William Q. Duong:** writing – original draft. **Ramola M. Panchal:** writing – original draft. **Ahmed M. Abou‐Zamzam:** writing – review and editing.

## Funding

The authors have nothing to report.

## Conflicts of Interest

The authors declare no conflicts of interest.

## Data Availability

Data sharing is not applicable to this article as no datasets were generated or analyzed during the current study.
